# Single Sensillum Recordings Reveal Antennal Responses of *Monochamus alternatus* and *Monochamus saltuarius* (Coleoptera: Cerambycidae) to Pheromones and Host Volatiles

**DOI:** 10.1007/s10886-026-01714-6

**Published:** 2026-04-18

**Authors:** Min-Jung Huh, Gwang-Hyun Roh, Kye-Chung Park, Il-Kwon Park

**Affiliations:** 1 Department of Agriculture, Forestry, and Bioresources, College of Agriculture and Life Sciences, Seoul National University, Seoul, 08826 Republic of Korea; 2 Research Corporation of University of Hawaii, University of Hawaii-Manoa, Honolulu, HI 96822 USA; 3 Department of Plant Medicine, Institute of Agriculture and Life Sciences, Gyeongsang National University, Jinju, Republic of Korea; 4 The New Zealand Institute for Bioeconomy Science, Christchurch Mail Centre, Private Bag 4704, Christchurch, 8140 New Zealand; 5 Research Institute of Agriculture and Life Science, College of Agriculture and Life Sciences, Seoul National University, Seoul, 08826 Republic of Korea

**Keywords:** Basiconic sensilla, Semiochemicals, Olfactory sensilla, Olfactory sensory neuron, Single sensillum recording

## Abstract

*Monochamus alternatus* Hope and *Monochamus saltuarius* Gebler (Coleoptera: Cerambycidae) are insect vectors of the pine wood nematode (PWN) on the Korean peninsula. Although they share an identical aggregation-sex pheromone, the two species exhibit different synergistic responses to host volatiles and other semiochemicals. In this study, we examined the electrophysiological responses of olfactory sensilla in both species to 22 semiochemicals, including pheromone, bark beetle pheromones, and host volatiles. Single-sensillum recordings (SSR) identified two distinct groups of basiconic-type olfactory sensilla, each associated with different functional groups. Sensilla basiconica A (SBaA) contained specialized olfactory sensory neurons (OSNs) that could be functionally distinguished into non-responsive, specialist like pheromone specialist, and generalist OSNs. In contrast, OSNs in sensilla basiconica B (SBaB) did not show clear functional classification into specific odor groups. Therefore, they were classified based on the number of compounds eliciting responses, as non-responsive neurons, specialists, and generalists. Notably, enantiomer-specific differences were observed among generalist OSNs. Most OSNs responded to both (+)- and (−)-*α*-pinene enantiomers, whereas a significantly higher proportion of OSNs showed exclusive responses to (−)-limonene compared to (+)-limonene. These results provide new insight into the olfactory mechanisms that underlie species-specific behavioral responses to semiochemicals in these economically important beetle vectors.

**Supplementary Information:**

The online version contains supplementary material available at 10.1007/s10886-026-01714-6.

## Introduction

*Monochamus alternatus* Hope (Coleoptera: Cerambycidae) and *M*. *saltuarius* Gebler are insect vectors of the pine wood nematode on the Korean Peninsula. Pine wilt disease has increased in Korea over the past five years, with approximately 1.49 million trees affected in 2025 and about USD 106 million allocated for its control, highlighting its substantial impact on pine forests (Korea Forest Service 2025).

Both species utilize 2-undecyloxy-1-ethanol (monochamol) as a male-produced aggregation-sex pheromone (Lee et al. 2017, 2018). Monochamol is known to act synergistically with host volatiles and bark beetle pheromones. In both species, attraction to monochamol increased when combined with *α*-pinene and ethanol (Lee et al. 2017, 2018). However, the two species differed in their response to bark beetle pheromones: *M*. *saltuarius* showed enhanced attraction to ipsenol or ipsdienol when presented together with monochamol, *α*-pinene, and ethanol (Lee et al. 2017), whereas *M*. *alternatus* did not (Lee et al. 2018). They showed differences in the synergistic effects of pheromone lures when combined with other pine host volatiles, such as *β*-caryophyllene, 3-carene, myrcene, *β*-pinene, and limonene (Lee et al. 2017, 2018). In contrast, when tested with *α*-pinene and limonene enantiomers, no differences in attraction were observed between enantiomers (Huh et al. 2025; Huh and Park 2025). These interspecific differences in olfactory attraction to semiochemicals may reflect variations in their underlying olfactory detection mechanisms.

On the cuticular surface of insects, there are structures known as sensilla, which function as receptors for external stimuli. Sensilla can be classified based on their morphology and distribution. By observing their structural characteristics, researchers can infer their sensory functions and the types of stimuli they detect, such as olfactory, gustatory, tactile, thermal, or humidity stimuli (Schneider 1964; Zacharuk and Shields 1991). Chemoreceptors are characterized by the presence of micropores on their surface, whereas mechanoreceptors possess a basal socket that detects hair movement and cuticular pressure. Chemoreceptors are generally divided into two functional groups based on micropore density location: uniporous chemosensilla, with one or a few micropores at the tip, and multiporous chemosensilla, with numerous micropores distributed across the surface (Zacharuk 1980). Uniporous chemosensilla are typically associated with gustatory function, while multiporous sensilla serve as olfactory receptors. The micropores provide direct contact between the external environment and the sensillum lymph, allowing odor molecules to diffuse inward or bind to odorant-binding proteins, which transport them to the dendrite terminals (Steinbrecht 1997).

Single sensillum recording (SSR) is widely used to analyze the responses of olfactory sensory neurons (OSNs) to volatile compounds. SSR provides higher resolution by recording the action potentials of individual OSNs within single sensilla, thereby revealing their response profiles to specific odorants (Olsson and Hansson 2013). To date, only two studies have employed SSR to investigate the function of olfactory sensilla in *Monochamus* spp. Dyer and Seabrook (1978) briefly examined *M. notatus* (Drury) and tested the responses of olfactory sensilla to *α*-pinene, limonene, and camphene, suggesting that *M. notatus* lacks specialized OSNs for detecting specific host volatiles. Following the discovery of the aggregation-sex pheromone of *M. galloprovincialis* (Olivier) (Pajares et al. 2010), Álvarez et al. (2015) analyzed the responses of olfactory sensilla in this species to 18 compounds across four categories: aggregation-sex pheromone, pine volatiles, bark beetle pheromone, and smoke volatiles. The OSNs present in sensilla basiconica were grouped into six clusters, each tuned to specific classes of volatiles or inhibited by certain compounds. However, no electrophysiological studies to characterize the responses of olfactory sensilla have yet been conducted in *M. alternatus* and *M. saltuarius*. In this study, we evaluated the responses of olfactory sensilla in the antennae of *M. alternatus* and *M. saltuarius* to 22 test compounds, including monochamol, ipsenol/ipsdienol, and 19 major host plant volatiles. Electrophysiological response profiles of antennal sensilla were analyzed by species and sex, and OSNs were classified into functional clusters. The enantiometric response profiles of their OSNs to *α*-pinene and limonene were also assessed in both species to explain the field assay results. This study not only provides new insights into the mechanism mediating species-specific olfactory communication but also enables the differential selection of candidate compounds for field attraction tests between the two species.

## Materials and Methods

### Insects

*Monochamus alternatus* and *M. saltuarius* used in experiments were obtained from OsangKinsect (Ye-san, Chungbuk, Republic of Korea). *Monochamus alternatus* were individually housed in 90 mm deli cups and provided with *Pinus densiflora* Siebold & Zucc. twigs and distilled water until use. They were reared at room temperature under a 16 L:8D photoperiod. Wet cotton balls were supplied to maintain the relative humidity at 40–50%. *Monochamus saltuarius* were reared using the same method, except they were supplied with *Pinus koraiensis* Siebold & Zucc. twigs as food. Adult beetles aged 5–20 days after sclerotization were used for SSR tests.

### Preparation of Test Compounds

Twenty-two compounds were prepared as test stimuli, including monochamol, bark beetle pheromones (ipsenol/ipsdienol), and host volatiles. Nineteen major host volatiles were selected, as they have been reported as major volatiles produced by *P. densiflora* and *P. koraiensis* (Kang et al. 2009; Son et al. 2015; Ahn et al. 2018; Park et al. 2021; Ji and Ji 2022). The sources and purities of test compounds are provided in Table 1.

**Table 1:** Compounds used for the single sensillum recording tests of *Monochamus alternatus* and *M. saltuarius*, with their purity, source, and references on dominant volatiles of *Pinus* spp

Mixture group	Compounds	CAS no.	Abbr.	Purity (%)	Source	Reference
Group 1	Monochamol	38471-47-5	PHER	98	KIP	-
	(+)-*α*-Pinene	7785-70-8	(+)-*α*-P	> 95	TCI	[1],2], [3], [4], [5]
	(-)-*α*-Pinene	7785-26-4	(-)-*α*-P	99	Sigma-Aldrich	[1], [2], [3], [4]
	*β*-Pinene	18172-67-3	*β*-P	> 94	TCI	[1], [2], [3], [4], [5]
	Ipsenol	14314-21-7	IPSE	93	Bedoukian	-
	Ipsdienol	14434-41-4	IPSD	95	Bedoukian	-
Group 2	(+)-Limonene	5989-27-5	(+)-L	97	Sigma-Aldrich	[1], [2], [3], [4]
	(-)-Limonene	5989-54-8	(-)-L	> 95	TCI	[1], [3]
	Myrcene	123-35-3	MY	> 75	Sigma-Aldrich	[1], [2], [3], [5]
	*β*-Caryophyllene	87-44-5	*β*-C	> 90	TCI	[3], [5]
	3-Carene	13466-78-9	3-C	95	Sigma-Aldrich	[1], [3]
	Ethanol	64-17-5	EtOH	99.9	Daejung	-
Group 3	Camphene	79-92-5	CAPE	> 99	Fluka	[3], [4], [5]
	*α*-Phellandrene	4221-98-1	*α*-PH	> 65	TCI	[1], [3], [5]
	Ocimene	13877-91-3	OC	> 90	SAFC	[3]
	*p*-Cymene	99-87-6	*p*-C	99	Sigma-Aldrich	[2], [3]
	Terpinolene	586-62-9	TE	> 85	TCI	[3], [4]
Group 4	Camphor	76-22-2	CAPO	96	Sigma-Aldrich	[3], [4]
	Sabinene	3387-41-5	SA	75	Sigma-Aldrich	[2], [3], [4]
	*α*-Terpineol	98-55-5	*α*-TEOL	> 95	TCI	[3], [4]
	*α*-Terpinene	99-86-5	*α*-TENE	> 85	Fluka	[2], [3], [4]
	*γ*-Terpinene	99-85-4	*γ*-TENE	97	Fluka	[2], [4]

[1] Kang et al. 2009; [2] Son et al. 2015; [3] Ahn et al. 2018; [4] Park et al. 2021; [5] Ji and Ji 2022

### SSR of Sensilla Basiconica

**Fig. 1:**
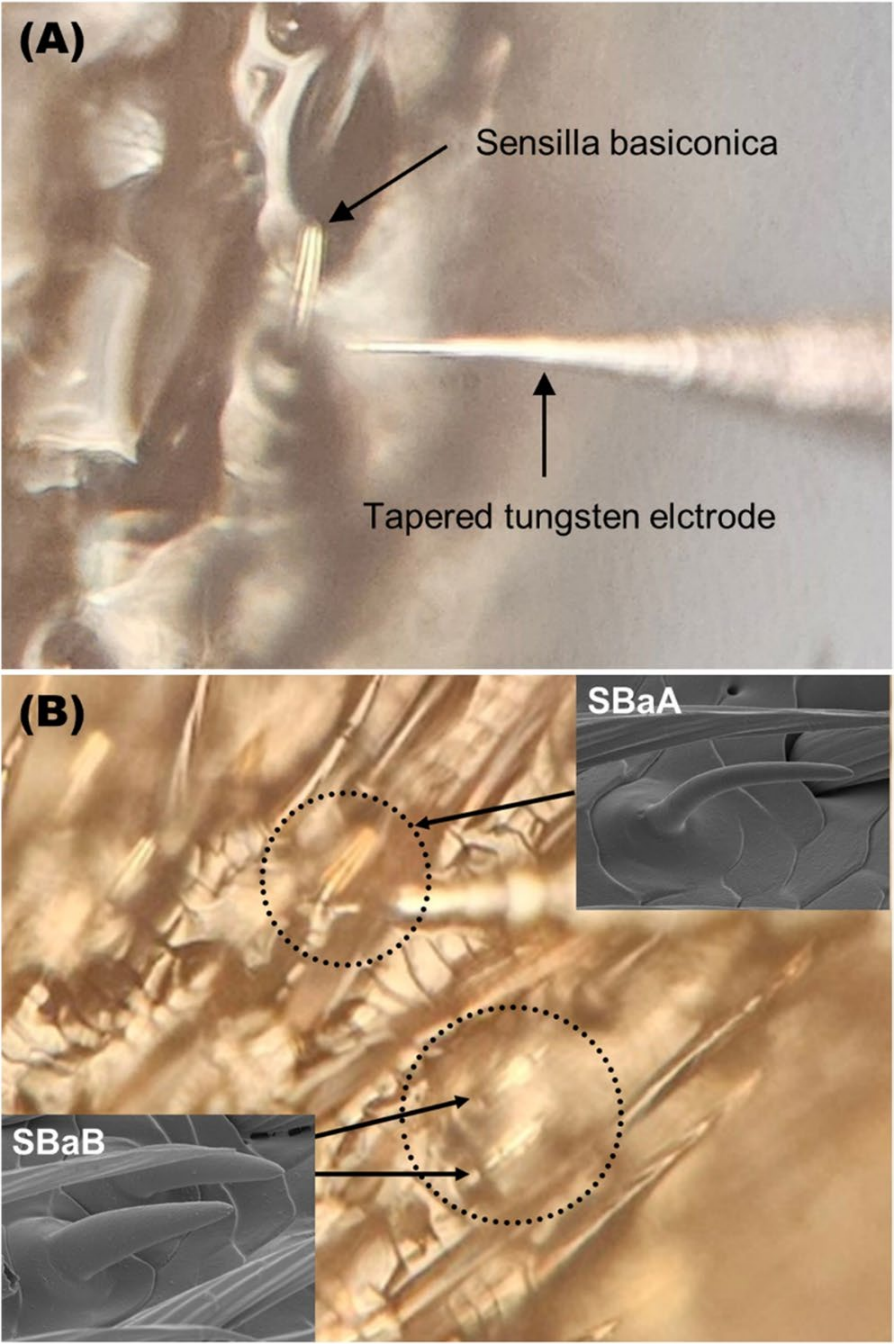
Light-Microscope image of a tungsten needle inserted into a sensillum at 500× (**A**). Comparison of light-microscopy and scanning electron microscope (SEM) images of sensilla basiconica A (SBaA) and basiconica B (SBaB) (**B**)

Individual beetles were mounted on a flat paraffin wax plate, with each body segment, leg, and antenna restrained using U-shaped copper wires (Fig. S1). The antennae were straightened and fixed so that the dorsal surface, where olfactory sensilla are most abundant, was oriented upward for optical microscopic observation. A stainless-steel needle (0.4 mm thickness) was inserted through the middle of the abdomen to serve as the reference electrode (Fig. S1). An electrochemically sharpened tungsten microelectrode (0.01 mm diameter, A-M Systems, Washington, USA) was used as the recording electrode, and its position was precisely controlled with a micromanipulator (Sutter Instruments, Novato, CA, USA). The right antenna was placed 1 cm from the center of a charcoal-filtered, humidified air stream passing through a cylindrical polystyrene tube. Antennal sensilla were observed under a compound microscope at 500× magnification (Fig. 1A). Signals from OSNs were amplified with an IDAC-4 (Syntech, Germany) and analyzed using Autospike software (ver.3.10, Syntech, Germany). The flow rates of both continuous and stimulus airflows were set at 3 L/min and were independently controlled by a stimulus controller (CS-55, Syntech, Germany).

For OSN characterization and clustering, each volatile compound was dissolved in hexane at a concentration of 10 µg/µl. Test solutions containing each test compound (22 compounds) were prepared, and four different mixture solutions (Table 1) were also prepared with the same concentration of each chemical at 10 µg/µl. 10 µl of each solution was applied to a piece of filter paper (3 × 20 mm), inserted into a Pasteur pipette after evaporating *n-*hexane for 10s, and used as a stimulus cartridge. The large end of the Pasteur pipette stimulus cartridge was sealed with aluminum foil when not in use. We replaced the cartridges after 5 times used. Control stimuli consisted of 10 µl *n-*hexane applied in the same manner.

Recordings were made from two basiconic sensilla types which were identified based on their morphological characteristics: thin sensilla basiconica (SbaA) and thick sensilla basiconica (Fig. 1B). For SSR, an electrochemically sharpened tungsten electrode was inserted into the base of a randomly selected sensillum to secure an electrical connection to its OSNs (Fig. 1B). Stable connection between the recording electrode and OSNs was confirmed by the presence of spontaneous spikes. A Pasteur pipette containing the test compound was inserted into the center of the glass tube, and odor stimuli were delivered as a 0.5-second air puff directed toward the antenna. Each stimulus was administered at intervals longer than 30 s to allow OSNs to recover to baseline activity. Electrophysiological responses were first tested with control (*n-*hexane), followed by stimulation with four mixtures of volatile compound groups listed in Table 1, tested in randomized order. If a significant change in spike frequency was detected in response to the mixture of the volatile group, the sensillum was subsequently tested with the individual compounds contained in the corresponding groups of volatiles. The order of test compounds was randomized.

In each recording, spikes were counted for 1 s before and after stimulation. When two groups of spikes with different amplitude, respectively, were detected, they were considered to originate from different OSNs and were counted separately (Fig. S2). For each OSN, the spike frequency in response to a test compound was calculated by subtracting the number of spikes before stimulation from that after stimulation. OSNs that responded significantly to fewer than five test compounds were classified as ‘specialist’, whereas those responding to multiple compounds were classified as ‘generalist’. OSNs that did not respond, which were defined as exhibiting fewer than 10 spikes in response to any test compound, were classified as ‘unresponsive’. SSR was conducted on seven males and seven females of *M. saltuarius* and *M. alternatus*.

### Statistical Analysis

The average responses of OSN classes to test compounds were analyzed using a generalized linear mixed model (GLMM) with negative binomial distribution and log link function, followed by Šídák’s post hoc test. Spike counts elicited within 1 s after stimulation were treated as the response variable, with test compound or doses as a fixed effect and individual OSN as a random effect. Significance of fixed effects was assessed using likelihood ratio tests (χ²). All statistical analyses were performed in R (ver. 4.3.1., R Development Core Team) using the packages ‘dplyr’, ‘lmerTest’, ‘glmmTMB’, DHARMa’, ‘car’, ‘emmeans’, ‘multcomp’, and ‘multcompView’.

## Results

### SSR of Sensilla Basiconica A and B

The numbers shown in Table 2 are the numbers of SBaA and SBaB sensilla tested in this study, and the numbers of sensilla responsive or non-responsive to the test compounds among the sensilla tested. Among the sensilla exhibiting spontaneous action potentials, some did not show any responses to the compounds. Such unresponsive sensilla were more prevalent in *M. saltuarius* than in *M. alternatus*, and more frequent in males than females. In *M. saltuarius*, 85.5% of SBaA and 41.8% of SBaB sensilla in males did not respond to the test compounds, whereas the proportion of unresponsive sensilla was lower in females, with 60.0% of SBaA and 27.0% of SBaB sensilla showing no responses. In *M. alternatus*, the overall proportion of unresponsive sensilla was lower than in *M*. *saltuarius*. In males, 55.3% of SBaA and 35.8% of SBaB were unresponsive, while in females, 27.9% of SBaA and 11.1% of SBaB sensilla showed no response to the tested compounds. Overall, eight different types of OSNs could be identified in SbaA and SBaB sensilla (Table 3). All eight types of ORNs were present in both sexes of both species.

**Table 2:** The number of respondents and non-respondents in two subtypes (SBaA and SBaB) of basiconic sensilla to the compounds tested in this study

Species	Sex	Sensillum type	No. of sensilla		
			Total	Responsive^1^	Non-responsive^2^
*M. saltuarius*	male (*n* = 7)	SBaA	83	12	71
		SBaB	43	25	18
	female (*n* = 7)	SBaA	45	18	27
		SBaB	37	27	10
*M. alternatus*	male (*n* = 7)	SBaA	56	25	31
		SBaB	39	25	14
	female (*n* = 7)	SBaA	43	31	12
		SBaB	27	24	3

^1^ Responsive sensilla exhibited more than 10 spontaneous spikes to at least one test compound

^2^ Non-responsive sensilla exhibited fewer than 10 spikes in response to all test compounds

**Table 3:** Numbers of olfactory sensory neurons (OSNs) and spike responses to each test compound by OSN functional type in sensilla basiconica A (SBaA) and basiconica B (SBaB) of *Monochamus alternatus* and *Monochamus saltuarius*

Sensillun type	OSN type	Responsiveness	Numbers observed				
			M. saltuarius			M. alternatus	
			Male	Female		Male	Female
SBaA	A1	Unresponsive	73	30		35	21
	A2	Monochamol specialist	7	6		9	8
	A3	Ipsenol/ipsdienol specialist	1	2		7	4
	A4	Specialist	1	5		2	7
	A5	Generalist	2	4		3	9
SBaB	B1	Unresponsive	25	14		20	7
	B2	Specialist	14	24		14	18
	B3	Generalist	15	12		16	16

### Clustering of OSNs in Sensilla Basiconica A (SBaA)

Type A1: Unresponsive

SBaA contained a large proportion of ‘unresponsive’ sensilla and OSNs (Table 3). These neurons elicited spontaneous spikes. Unresponsive OSNs were also observed in the sensilla containing OSN responsive to test compounds. When more than one OSN is co-compartmentalized in a sensillum, one OSN responded to specific volatiles, whereas the other OSN remained unresponsive in some cases. Among OSNs in SBaA sensilla, the proportions of type A1 OSNs were 62.5% and 42.9% in males and females of *M*. *alternatus*, respectively, and 86.9% and 63.8% in males and females of *M*. *saltuarius*, respectively.

#### Type A2: Monochamol Specialist

**Fig. 2:**
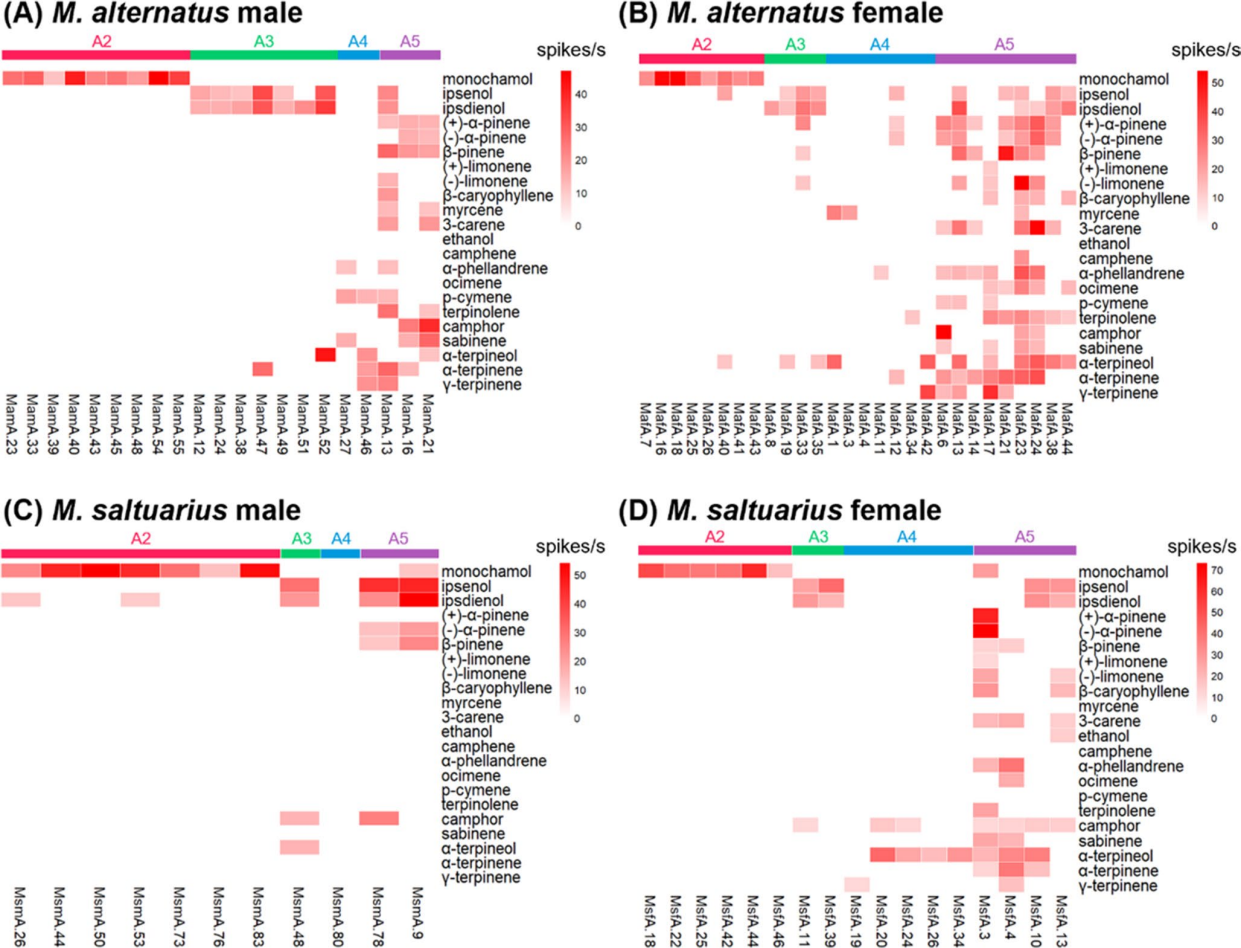
Heatmaps of olfactory sensory neuron (OSN) responses recorded from sensilla basiconica type A (SBaA) in *Monochamus alternatus* males (**A**), females (**B**), and *Monochamus saltuarius* males (**C**) and females (**D**). Rows are tested semiochemicals; columns are individual OSNs. Color intensity indicates firing frequency (spikes/s)

**Fig. 3:**
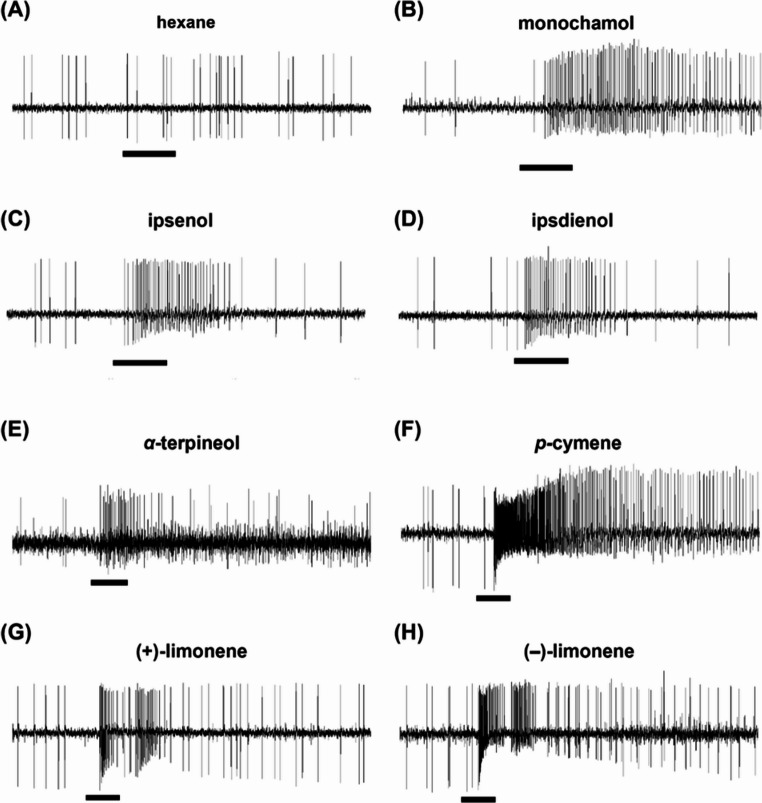
Response profiles of olfactory sensory neurons (OSNs) to test compounds. The black bar indicates the 0.5-second stimulus. Representative spike activity to the negative control (*n-*hexane) (**A**). A2-type OSN in a female *Monochamus saltuarius* responding to 100 µg monochamol (**B**). A3-type OSN in a male *M. saltuarius* responding to 100 µg ipsenol (**C**) and ipsdienol (**D**). A4-type OSN in a female *M. saltuarius* responding to 100 µg *α*-terpineol (**E**). B3-type OSN in a male *M. saltuarius* responding to 100 µg *p*-cymene (**F**), (+)-limonene (**G**), and (–)-limonene (**H**). Comparable response profiles were observed in *Monochamus alternatus*

The second clustered functional type of OSNs, type A2, responded exclusively to monochamol with high sensitivity, and was therefore designated as a ‘monochamol specialist’ (Figs. 2 and 3B). In *M. alternatus*, 16.1% of male OSNs (Fig. 2A: MamA.23, 33, 39, 40, 43, 45, 48, 54, 55) and 16.3% of female OSNs (Fig. 2B: MafA.7, 16, 18, 25, 26, 40, 41, 43) were classified as type A2. In contrast, *M. saltuarius* exhibited a lower proportion of type A2 OSNs, with 8.3% in males (Fig. 2C: MsmA.26, 44, 50, 53, 73, 76, 83) and 12.8% (Fig. 2D: MsfA.18, 22, 25, 42, 44, 46) in females.

#### Type A3: Ipsenol and Ipsdienol Specialist

The third functional type of OSNs, type A3, clustered based on their response profiles, was designated as the ‘ipsenol and ipsdienol specialist’. These OSNs showed pronounced responses to ipsenol and ipsdienol (Figs. 2 and 3C, and 3D). In *M*,* alternatus*, 12.5% of male OSNs (Fig. 2A: MamA.12, 24, 38, 47, 49, 51, 52) and 8.2% female OSNs (Fig. 2B: MafA.8, 19, 33, 35) were categorized as type A3. Some of type A3 OSNs exhibited exclusive responses to ipsenol and ipsdienol, whereas some other type A3 OSNs responded to a few other host volatiles such as *α*-terpineol and *α*-terpinene. In *M. saltuarius*, 1.2% of male OSNs (Fig. 2C: MsmA.48) and 4.3% of female OSNs (Fig. 2D: MafA.11, 39) were classified as type A3. Although primarily responsive to ipsenol and ipsdienol, these type A3 OSNs also responded to one or two host volatiles, such as camphor and *α*-terpineol (Fig. 2).

#### Type A4: Specialist

Among the OSNs in SBaA, some responded specifically to fewer than five host volatiles (Figs. 2 and 3E). These were categorized as the ‘specialist’ type A4 OSNs. In female *M. saltuarius*, type A4 OSNs were predominantly *α*-terpineol specialists (Fig. 2): four OSNs (MsfA.20, 24, 26, 34) with exclusive responses to *α*-terpineol were identified in females, whereas only one OSN (MsmA.80) that responded solely to *α*-terpineol was found in males. In female *M. alternatus*, seven type A4 OSNs responded to *α*-terpineol, *β*-caryophyllene, *γ*-terpinene, terpinolene, *α*-phellandrene, or *α*-pinene with *α*-terpinene. Type A4 OSNs were more frequently observed in females than in males. In *M. alternatus*, only two OSNs (3.6%) of this type were found in males (Fig. 2A: MamA.27, 46), whereas 14.3% were observed in females (Fig. 2B: MafA.1, 3, 4, 11, 12, 34, 42). Similarly, in *M. saltuarius*, A4 OSNs accounted for 1.2% in males (Fig. 2C) and 10.6% in females (Fig. 2D: MsfA.19, 20, 24, 26, 34). Distinct interspecific differences were also observed in the responses of type A4 OSNs to host volatile compounds. *β*-Caryophyllene–specialist OSNs were detected only in females of *M. alternatus* and not in females of *M. saltuarius*, whereas camphor-specialist OSNs were present in *M. saltuarius* but absent in *M. alternatus*.

#### Type A5: Generalist

The remaining OSNs exhibited consistent responses to multiple compounds. These neurons were classified as type A5 ‘generalist’ OSNs. This type of OSNs was more frequently observed in females than in males. In *M. alternatus*, three OSNs (5.4%) (Fig. 2A: MamA.13, 16, 21) were detected in males, whereas nine OSNs (18.4%) (Fig. 2B: MafA.6, 13, 14, 17, 21, 23, 24, 38, 44) were detected in females. In *M. saltuarius*, type A5 OSNs accounted for 2.4% (Fig. 2C: MsmA.9, 78) in males and 8.5% (Fig. 2D: MsfA.3, 4, 10, 13) in females.

Some neurons demonstrated broader sensitivity, responding to more than five volatiles, while showing enhanced sensitivity to specific one or two. In *M. alternatus*, a single A5 OSN (MamA.21) was identified in males, displaying high sensitivity to camphor with a spike frequency of 39 (Fig. 2). In females, five A5 OSNs (MafA.6, 17 21, 23, 24) were identified, each exhibiting elevated responses to particular compounds, including camphor, *β*-pinene, (–)-limonene, *γ*-terpinene, or 3-carene (Fig. 2). In *M*. *saltuarius*, one OSN (MsfA.3) in females responded with 10–30 spikes to 12 compounds, but showed markedly higher activity (> 60 spikes) to both (+)-*α*-pinene and (–)-*α*-pinene (Fig. 2).

### Average Responses of A-type OSNs to the Test Compounds

**Fig. 4:**
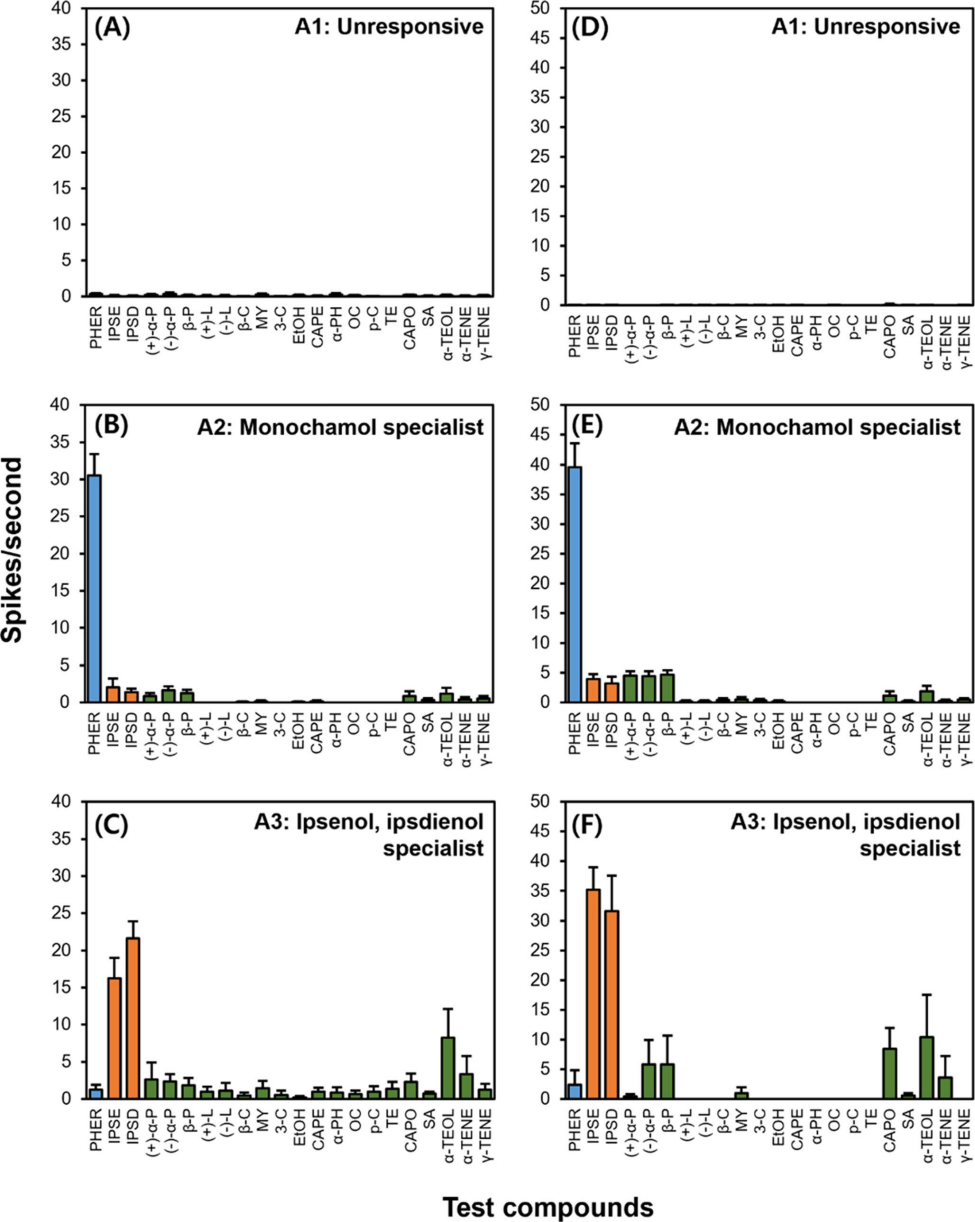
Mean response of A1–A3 olfactory sensory neuron (OSN) types to 100 µg of each test compound in *Monochamus alternatus* (**A–C**) and in *Monochamus saltuarius* (**D–F**). Colors denote volatile categories (blue, aggregation-sex pheromone; orange, bark beetle pheromone; green, host volatiles; abbreviations as in Table 1). Mean spike numbers (± SE) and statistics are provided in Supplementary Tables S1–S2

Average response intensities of A-type OSNs to test compounds are shown in Fig. 4 and supplementary materials (Fig. S3, Tables S1 and S2). Among A-type OSNs, the monochamol specialist neurons (A2) of *M. alternatus* (30.5 ± 2.9 spikes/s) and *M. saltuarius* (39.5 ± 4.0 spikes/s) responded strongly and selectively to monochamol (*M. alternatus*: χ² = 178.33, df = 21, *p* < 0.001; *M. saltuarius*: χ² = 456.66, df = 21, *p* < 0.001). Other test compounds that showed less than 10 spikes/s were grouped separately from those that did not elicit any OSN responses (Fig. 4B and E; Tables S1 and S2). Similarly, ipsenol/ipsdienol-specialist OSNs (A3) in both *Monochamus* species displayed strong and exclusive responses to their respective ligands, compared to other compounds except *α*-terpineol (*M. alternatus*: χ² = 238.54, df = 21, *p* < 0.001; *M. saltuarius*: χ² = 34.145, df = 21, *P* < 0.001) (Fig. 4C and F). Although unresponsive OSNs (A1) in *M. alternatus* showed statistically significant differences in spike frequency across compounds, the spike rates were all below 10 spikes/s and thus considered functionally unresponsive (Fig. 4A, Tables S1). For unresponsive OSNs in *M. saltuarius*, statistical comparison was not performed due to the high frequency of zero spike responses in the dataset (Fig. 4D, Tables S2). Overall, these results indicate that the monochamol- and ipsenol/ipsdienol-specific OSNs are highly tuned to their target semiochemicals, whereas unresponsive OSNs remain insensitive to all tested volatiles. Averaged response intensities of host volatile-specialists (A4) and generalists (A5) are presented in Fig. S3, Tables S1 and S2. In both *M. alternatus* and *M. saltuarius*, A4-type OSNs exhibited only weak activities to most compounds, but showed significant responses to certain host volatile compounds like *α*-terpineol, which elicited responses exceeding 10 spikes/s (*M. alternatus*: χ² = 69.951, df = 21, *p* < 0.001; *M. saltuarius*: χ² = 139.25, df = 21, *p* < 0.001). Conversely, A5-type OSNs responded to a broad array of test compounds, with more than 5 terpene compounds such as *α*-pinene, *β*-pinene, terpinolene, and *α*-terpineol inducing responses greater than 10 spikes/s in both species (*M. alternatus*: χ² = 3.8574, df = 21, *p* < 0.001; *M. saltuarius*: χ² = 25.922, df = 21, *p* = 0.2094).

### Clustering of OSNs in Sensilla Basiconica B (SBaB)

The clustering of SBaB OSNs was particularly challenging owing to their broad and heterogeneous response profiles across multiple compounds. To resolve this, OSNs were classified according to the number of compounds that evoked spiking activity and subsequently designated as either ‘specialist’ or ‘generalist’ OSNs.

### Type B1: Unresponsive

Similar to type A1, certain sensilla and OSNs in SBaB showed no responses to the test compounds while exhibiting spontaneous spiking activities (Table 3). These neurons were classified as type B1 and designated as ‘unresponsive’. One or two OSNs could coexist within a single sensillum, and type B1 OSNs were frequently observed in association with responsive OSNs in the same sensillum. In *M. alternatus*, 40.0% of male OSNs were classified as type B1, compared with 17.1% in females (Table 3). In *M. saltuarius*, the proportion of type B1 OSNs was 46.3% in males and 28.0% in females.

### Type B2: Specialists

**Fig. 5:**
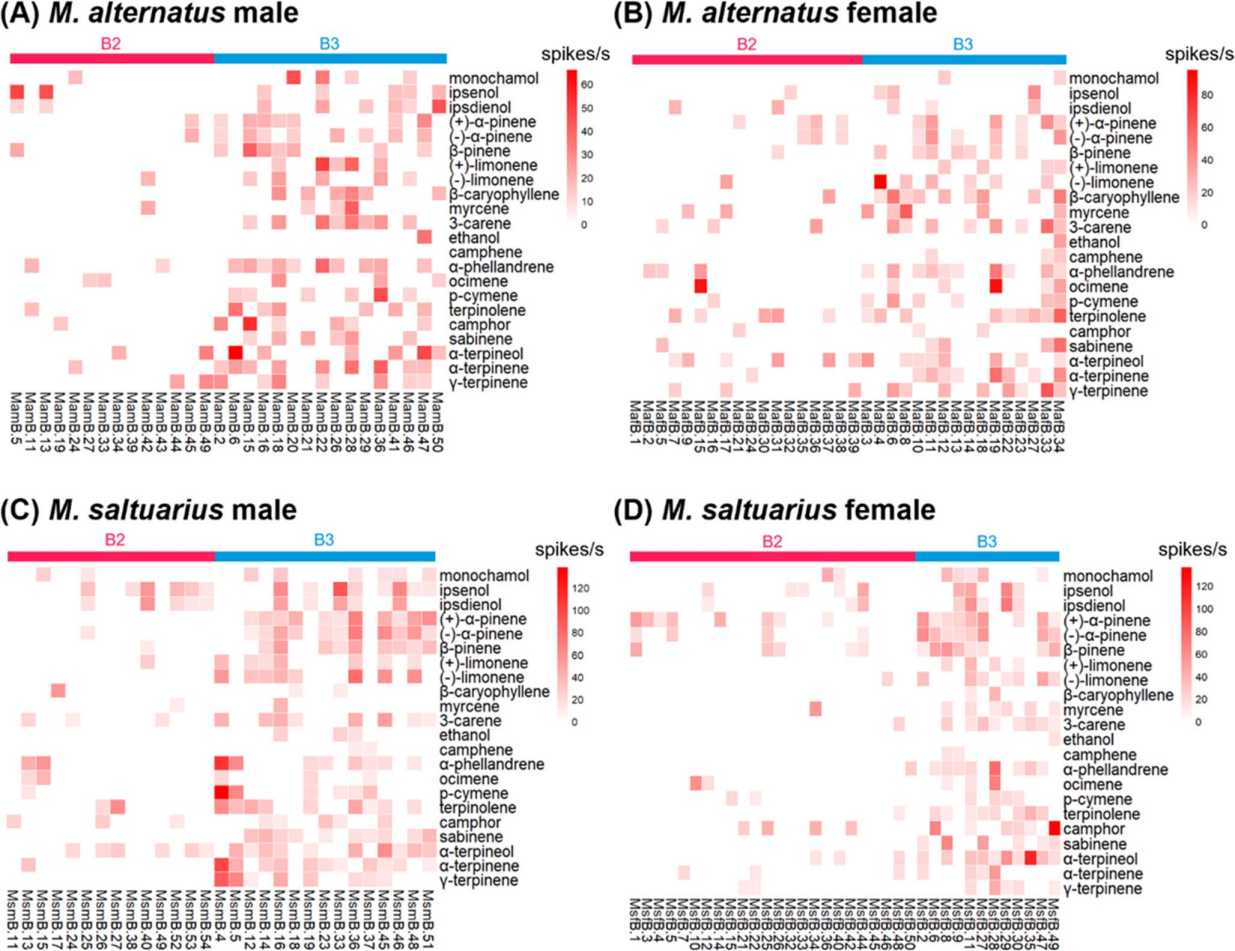
Heatmaps of olfactory sensory neuron (OSN) responses recorded from sensilla basiconica type B (SBaB) in *Monochamus alternatus* males (**A**), females (**B**), and *Monochamus saltuarius* males (**C**) and females (**D**). Rows are tested semiochemicals, and columns are individual OSNs. Color intensity indicates firing frequency (spikes/s)

Unlike the specialist OSNs of SBaA, those in SBaB were difficult to cluster into distinct groups. These OSNs typically responded to one to four test compounds but displayed no consistent patterns. They were classified as type B2 and designated as ‘specialists.’ Some OSNs exhibited response profiles resembling those of the SBaA specialists, whereas others responded to only one or two host volatiles without similarity to SBaA patterns. For instance, one OSN responded exclusively to ipsenol, ipsdienol, and *α*-terpineol, similar to type A3, while another responded solely to *α*-phellandrene or myrcene (Fig. 5), which were not detected in SBaA. Among the OSNs in SBaB of *M. alternatus*, 28.0% in males (Fig. 5A: MamB.5, 11, 13, 19, 24, 27, 33, 34, 39, 42, 43, 44, 45, 49) and 43.9% in females (Fig. 5B: MafB.1, 2, 5, 7, 9, 15, 16, 17, 21, 24, 30, 31, 32, 35, 36, 37, 38, 39) were classified as type B2. In *M. saltuarius*, type B2 OSNs accounted for 25.9% in males (Fig. 5C: MsmB.11, 13, 15, 17, 24, 25, 26, 27, 38, 40, 49, 52, 53, 54) and 48.0% in females (Fig. 5D: MsfB.1, 3, 4, 5, 7, 10, 12, 14, 15, 21, 22, 25, 26, 32, 33, 34, 36, 40, 42, 44, 45, 46, 48, 50).

### Type B3: Generalist

Similar to type A5, the generalist OSNs (type B3) of SBaB responded to multiple volatiles. Type B3 OSNs typically produced an increase of 20–30 spikes in response to most volatiles (Fig. 3F, G & H). In *M. alternatus*, type B3 OSNs accounted for 32.0% in males (Fig. 5A: MamB.2, 6, 15, 16, 18, 20, 21, 22, 26, 28, 29, 36, 41, 46, 47, 50) and 39.0% in females (Fig. 5B: MafB.3, 4, 6, 8, 10, 11, 12, 13, 14, 18, 19, 22, 23, 27, 33, 34), whereas in *M. saltuarius*, they represented 27.8% in males (Fig. 5C: MsmB.4, 5, 12, 14, 16, 18, 19, 23, 33, 36, 37, 45, 46, 48, 51) and 24.0% in females (Fig. 5D: MsfB.2, 6, 8, 9, 11, 27, 28, 29, 30, 35, 47, 49).

Although SBaB OSNs were responsive to many compounds, some of them responded strongly to one or two specific compounds. For example, an OSN in a female *M. alternatus* elicited 12–25 spikes in response to four compounds but 95 spikes to (–)-limonene (MafB.4 in Fig. 5). These OSNs differed from SBaA specialists, which typically responded to fewer than three compounds, by showing activity to multiple compounds. However, they were also distinct from other ‘generalists’, as they exhibited disproportionately high responses to one or two specific compounds. The compounds eliciting strong responses varied among OSNs, with more than 80 spikes recorded in response to ocimene, (–)-limonene, *α*-terpineol, or camphor. The maximum response observed was 138 spikes, elicited by *p*-cymene in a male *M*. *saltuarius* (MsmB.4 in Fig. 5).

### Average Responses of B-type OSNs to the Tested Compounds

**Fig. 6:**
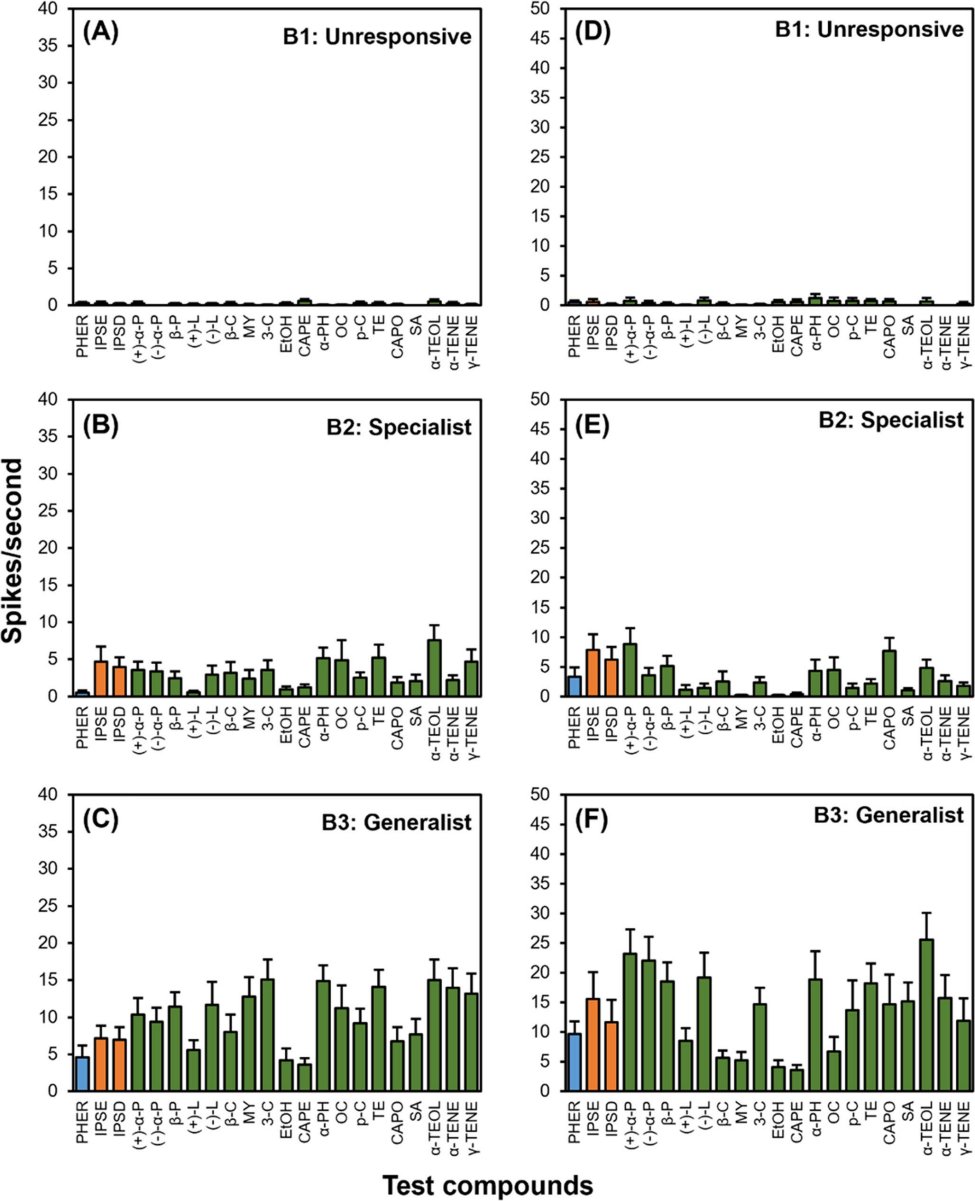
Mean response of B1–B3 OSN types to 100 µg of each test compound in *Monochamus alternatus* (**A–C**) and in *Monochamus saltuarius* (**D–F**). Colors denote volatile categories (blue, aggregation-sex pheromone; orange, bark beetle pheromone; green, host volatiles; abbreviations as in Table 1). Mean spike numbers (± SE) and statistics are provided in Supplementary Tables S1–S2

Average response intensities of B-type OSNs to compounds tested are shown in Fig. 6 and supplementary materials (Tables S1 and S2). In both *M*. *alternatus* and *M*. *saltuarius*, B2-type OSNs (specialist) exhibited relatively weak responses to most compounds (Fig. 6B and E), except for *α*-terpineol in *M*. *alternatus* and (+)-*α*-pinene in *M*. *saltuarius*, which elicited spike numbers as 7.6 ± 2.1 spikes/s and 8.8 ± 2.7 spikes/s, respectively (*M. alternatus*: χ² = 90.91, df = 21, *p* < 0.001; *M. saltuarius*: χ² = 167.21, df = 21, *p* < 0.001). Similar to A5-type OSNs, B3-type OSNs (generalist) responded to a broad range of host volatiles inducing strong excitatory responses in both species (*M. alternatus*: χ² = 77.726, df = 21, *p* < 0.001; *M. saltuarius*: χ² = 114.71, df = 21, *p* < 0.001) (Fig. 6C and F). In contrast, unresponsive OSNs (B1) showed very low spike frequencies across all tested compounds (Fig. 6A and D).

### SSR Responses to Enantiomers of *α*-pinene and Limonene.

In field assays (Huh et al. 2025; Huh and Park 2025), *M. alternatus* and *M. saltuarius* did not show significant differences in attraction between enantiomers of *α*-pinene or limonene when combined with pheromone traps. To further investigate potential stereospecific effects at the neuronal level, enantiomeric compounds were analyzed separately to determine whether neurons responsive to each enantiomer exhibited distinct response patterns to other tested compounds. However, no clear clustering of neurons was observed based on differential responses to enantiomers in relation to other test compounds.

. OSN responses to enantiomers of *α*-pinene and limonene varied among individual neurons. For *α*-pinene, most responsive OSNs reacted to both enantiomers (Table 4). OSNs that responded exclusively to (+)-*α*-pinene were more abundant in females than those responding to (–)-*α*-pinene only. For limonene, OSNs responsive to (–)-limonene only were more common in females than OSNs responsive to both enantiomers or (+)-limonene only (Table 4). The strongest response observed was 95 spikes to (–)-limonene in a female *M. saltuarius* (MafB.4), whereas the highest response to (+)-limonene was 49 spikes in a male *M. alternatus* (MamB.22) (Fig. 5).

**Table 4:** Numbers of olfactory sensory neurons responsive to the enantiomers of *α*-pinene and limonene

Compound	Response	M. saltuarius			M. alternatus	
		male	female		male	female
*α*-pinene	(+)-*α*-pinene	2	6		0	5
	(–)-*α*-pinene	3	2		4	1
	both	11	12		12	15
limonene	(+)-limonene	1	2		2	3
	(–)-limonene	2	8		4	10
	both	7	3		3	4

## Discussion

This study, to our knowledge, provides the first OSN-level electrophysiological characterization of olfactory responses in *M*. *alternatus* and *M*. *saltuarius*. Clear species- and sex-specific differences in the responses of *M. alternatus* and *M. saltuarius* OSNs to pheromones and host volatiles were demonstrated in this study. The two subtypes, SBaA and SBaB, were functionally distinguished based on their olfactory responses to semiochemicals.

In both species, OSNs housed in SBaA were predominantly specialists responsive to a narrow range of volatile compounds. The OSNs in SBaA are therefore likely to play a crucial role in the chemical communication of *M*. *alternatus* and *M*. *saltuarius*, in association with respective olfactory-active compounds that are essential for the ecology of both species. Among these specialized OSNs, type A2 OSNs present in both sexes of both species responded exclusively to monochamol, an aggregation-sex pheromone common to several *Monochamus* species (Pajares et al. 2010; Teale et al. 2011; Fierke et al. 2012; Lee et al. 2017, 2018). Similarly, the presence of OSNs specialized for monochamol has also been reported in *M*. *galloprovincialis* (Álvarez et al. 2015). *Monochamus galloprovincialis* shares similar morphological features of antennal sensilla basiconica with *M. alternatus* and *M. saltuarius*. In all three species, two types of sensilla basiconica with multiporous surfaces were identified as olfactory sensilla (Álvarez et al. 2015; Huh et al. 2023). The occurrence of monochamol-specific OSNs in *M*. *alternatus*,* M*. *saltuarius*, and *M*. *galloprovincialis* strongly suggests that this sensory specialization is conserved across the genus *Monochamus*. Given that monochamol functions as an aggregation-sex pheromone in multiple congeners, it is reasonable to infer that other species utilizing monochamol as a pheromone are also likely to possess OSNs narrowly tuned to this compound. These findings highlight the evolutionary significance of monochamol detection in the chemical communication system of *Monochamus* beetles.

Beyond the pheromone-tuned channels, SBaA contained host-volatile specialists (Type A4) that were narrowly tuned—most prominently to *α*-terpineol, with some OSNs specialized to *β*-caryophyllene or camphor. The female-biased presence of type A4 OSNs in both species (*M. alternatus*: 14.3% in females vs. 3.6% in males; *M. saltuarius*: 10.6% vs. 1.2%) seems to be associated with a greater female investment in host assessment (e.g., oviposition-site recognition). Field tests with *α*-terpineol and camphor will be valuable to link these peripheral profiles to attraction and decision-making. By contrast, Type A5 OSNs were broadly tuned (10–30 spikes across multiple host volatiles) yet often showed elevated sensitivity to one or two compounds. A5 also exhibited a female bias (*M. alternatus*: 18.4% vs. 5.4%; *M. saltuarius*: 8.5% vs. 2.4%), suggesting roles in host localization under variable odor plumes. Notably, the functional profile of A5 OSNs resembles that of the *α*-pinene/generalist OSNs described in *M*. *galloprovincialis*, which responded broadly to multiple compounds but showed enhanced sensitivity to *α*-pinene (Álvarez et al. 2015). Such parallels suggest that broadly tuned OSNs with biased sensitivity may represent one sensory strategy used by *Monochamus* species for host localization under heterogeneous odor environments. At the same time, we cannot exclude the alternative possibility that some OSNs that appeared broadly tuned to our panel of 22 compounds are in fact highly specialized for other, ecologically relevant ligands that were not included in our stimulus set. In this context, future dose–response analyses with an expanded panel of host and non-host volatiles would be particularly valuable for refining the interpretation of peripheral sensitivity profiles.

OSNs in SBaB also included both specialists and generalists; but they could not be clustered further into distinct groups due to the lack of consistent response patterns to the test compounds. Among the generalists, some OSNs exhibited significantly strong responses to one or a few compounds, like the type A5 OSN of SBaA in this study and *α*-pinene/generalist OSNs described in *M*. *galloprovincialis* (Álvarez et al. 2015). As these OSNs could not be further subdivided, additional studies are needed to characterize their spike patterns more precisely.

Ethanol did not elicit strong responses in the subset of OSNs we recorded from in our SSR experiments. Nevertheless, ethanol is a well-known kairomone released by stressed trees and is widely incorporated into pheromone lures for *Monochamus* species (Miller 2006; Lee et al. 2017, 2018; Dong et al. 2024). Consistently, Álvarez et al. (2015) also tested ethanol in SSR recordings with *M. galloprovincialis* and found no evidence of OSNs that were clearly specialized for ethanol. Although these SSR data from different *Monochamus* species do not support the presence of ethanol-specific OSNs, they are based on recordings from a limited subset of sensilla and therefore cannot rule out the existence of dedicated ethanol-responsive neurons in the antennae. One possible interpretation is that ethanol is detected primarily by broadly tuned OSNs and acts as a general host-stress cue that modulates responses to other volatiles, which could help explain its relatively weak electrophysiological responses despite its well-documented ecological importance as a kairomone. However, more comprehensive sampling of sensilla combined with dose–response analyses will be required to evaluate this hypothesis.

Our study indicates the presence of enantiospecific OSNs for *α*-pinene and limonene in the antennae of *M*. *alternatus* and *M*. *saltuarius*. The responses of OSNs to *α*-pinene enantiomers varied, in which some neurons responded similarly to both (+)- and (–)-*α*-pinene, while others exhibited stronger or exclusive responses to one enantiomer. In the case of limonene, OSNs strongly responsive to (−)-limonene were commonly observed, whereas OSNs selective to (+)-limonene were rare in both species. Although these results suggest the involvement of olfactory receptors in distinguishing different enantiomers, however, field experiments showed no significant differences in behavioral attractions to the enantiomers of *α*-pinene or limonene (Huh et al. 2025; Huh and Park 2025). Taken together, these electrophysiological and field studies may indicate that such enantiomeric discrimination of OSNs is not necessarily translated into divergent behavioral outcomes, although OSNs are capable of discriminating between enantiomers. This highlights that fine-scale discrimination at the receptor level may have a limited influence on behavioral outcomes, where multiple cues interact simultaneously. Consistent with this interpretation, Miller et al. (2024) suggested that host-plant enantiomers are not critical determinants of bark beetle attraction, as responses varied across species and locations regardless of the dominant host enantiomer. Collectively, these findings demonstrate that enantiomer-specific OSN tuning is not always predictive of species-level behavioral responses, which are more likely determined by higher-order integration and ecological context. Furthermore, the fact that these *Monochamus* species retain enantiomer-specific OSNs even if they no longer actively use such fine enantiomeric discrimination suggests that these peripheral channels may either subserve additional, as yet unidentified functions or represent sensory legacies of past selection pressures.

In conclusion, this study provides the first detailed electrophysiological characterization of OSNs in *M. alternatus* and *M. saltuarius*. Both species shared several conserved traits, including the presence of monochamol-specific OSNs across sexes, a parallel sensillar organization in which SBaA housed predominantly specialists and SBaB predominantly generalists, and the coexistence of narrowly tuned and broadly responsive OSNs for host volatiles. At the same time, they exhibited clear species-specific differences in the relative abundance and sensitivity of host-volatile OSNs, in the female-biased incidence of certain OSN types, and in their behavioral responses to ipsenol and ipsdienol. These contrasting patterns highlight that while some olfactory features are evolutionarily conserved within the genus *Monochamus*, others have diverged in response to ecological pressures and sex-specific roles. Collectively, these findings advance our understanding of olfactory specialization and diversification in the genus *Monochamus* and provide valuable insights for optimizing pheromone-based monitoring and pest management strategies.

## Supplementary Information

Below is the link to the electronic supplementary material.

Supplementary Material 1

## Data Availability

The datasets generated and/or analyzed during the current study are available from the corresponding author upon reasonable request.
