# Supplementary material for: Single Sensillum Recordings Reveal Antennal Responses of Monochamus alternatus and Monochamus saltuarius (Coleoptera: Cerambycidae) to Pheromones and Host Volatiles
Source: J Chem Ecol. 2026 Apr 18;52(3):36. doi: 10.1007/s10886-026-01714-6 (PMC13091885; doi:10.1007/s10886-026-01714-6)
Supplement: Supplementary file 1 — Supplementary Material 1 [file 10886_2026_1714_MOESM1_ESM.docx]

**Table S1.** Mean spike numbers (± SE) for sensilla basiconica A (SbaA) and sensilla basiconica B (SBaB) olfactory sensory neurons (OSNs) of *Monochamus* *alternatus* responding to pheromones and host volatiles

| Compounds | SBaA | | | | |  | SBaB | | |
| --- | --- | --- | --- | --- | --- | --- | --- | --- | --- |
|  | A1 | A2 | A3 | A4 | A5 |  | B1 | B2 | B3 |
|  | 56^1^ | 17 | 11 | 9 | 12 |  | 27 | 32 | 32 |
| monochamol | 0.3±0.2^a2^ | 30.5±2.9^b^ | 1.3±0.6^a^ | 0^a^ | 2.1±1.1^a^ |  | 0.3±0.2^a^ | 0.5±0.3^a^ | 4.6±1.5^ab^ |
| ipsenol | 0.1±0.1^a^ | 2.1±1.2^a^ | 16.3±2.7^cd^ | 2.0±2.0^a^ | 9.5±2.5^cd^ |  | 0.3±0.2^a^ | 4.7±2.1^ab^ | 7.2±1.7^abc^ |
| ipsdienol | 0.1±0.1^a^ | 1.4±0.5^a^ | 21.6±2.3^d^ | 0.6±0.6^a^ | 12.8±3.3^cd^ |  | 0.2±0.1^a^ | 4.0±1.3^ab^ | 7.0±1.7^abc^ |
| (+)-*α*-pinene | 0.2±0.2^a^ | 0.9±0.4^a^ | 2.6±2.2^a^ | 1.4±1.4^a^ | 17.0±3.0^d^ |  | 0.3±0.3^a^ | 3.6±1.1^ab^ | 10.4±2.2^abc^ |
| (-)-*α*-pinene | 0.4±0.2^a^ | 1.6±0.5^a^ | 2.4±0.9^ab^ | 1.8±1.8^a^ | 14.3±2.8^cd^ |  | 0.0±0.0^a^ | 3.4±1.2^ab^ | 9.4±1.9^abc^ |
| *β*-pinene | 0.2±0.1^a^ | 1.2±0.5^a^ | 1.8±1.0^ab^ | 2.4±1.6^a^ | 18.1±4.2^d^ |  | 0.2±0.1^a^ | 2.5±0.9^ab^ | 11.4±2.0^bc^ |
| (+)-limonene | 0.1±0.1^a^ | 0^ab^ | 1.0±0.7^a^ | 2.3±0.9^a^ | 5.1±1.0^abcd^ |  | 0.1±0.1^a^ | 0.6±0.2^a^ | 5.6±1.3^abc^ |
| (-)-limonene | 0.1±0.1^a^ | 0^ab^ | 1.1±1.1^a^ | 2.0±0.9^a^ | 13.3±4.2^cd^ |  | 0.2±0.2^a^ | 2.9±1.2^ab^ | 11.7±3.1^bc^ |
| *β*-caryophyllene | 0.1±0.0^a^ | 0.1±0.1^a^ | 0.5±0.4^a^ | 6.8±3.8^a^ | 5.1±1.5^abcd^ |  | 0.2±0.2^a^ | 3.2±1.4^ab^ | 8.0±2.4^abc^ |
| myrcene | 0.3±0.1^a^ | 0.1±0.1^a^ | 1.5±1.0^ab^ | 2.1±1.2^a^ | 7.4±2.3^abcd^ |  | 0.1±0.1^a^ | 2.4±1.2^ab^ | 12.8±2.6^bc^ |
| 3-carene | 0.1±0.1^a^ | 0^ab^ | 0.5±0.5^a^ | 2.8±1.2^a^ | 17.1±4.2^cd^ |  | 0.1±0.1^a^ | 3.6±1.3^ab^ | 15.1±2.7^c^ |
| ethanol | 0.2±0.1^a^ | 0.1±0.1^a^ | 0.2±0.2^a^ | 0.5±0.5^a^ | 1.8±0.5^ab^ |  | 0.3±0.2^a^ | 1.0±0.4^ab^ | 4.2±1.6^a^ |
| camphene | 0.1±0.1^a^ | 0.1±0.1^a^ | 1.0±0.5^a^ | 0.5±0.2^a^ | 4.5±1.9^abc^ |  | 0.6±0.3^a^ | 1.2±0.4^ab^ | 3.6±0.9^a^ |
| *α*-phellandrene | 0.3±0.2^a^ | 0^ab^ | 0.8±0.7^a^ | 5.0±1.7^a^ | 13.4±3.0^cd^ |  | 0.1±0.1^a^ | 5.1±1.5^ab^ | 14.9±2.1^c^ |
| ocimene | 0.1±0.1^a^ | 0^ab^ | 0.6±0.5^a^ | 2.6±1.1^a^ | 9.1±2.3^bcd^ |  | 0.1±0.1^a^ | 4.9±2.7^ab^ | 11.2±3.1^abc^ |
| *p*-cymene | 0^a^ | 0^ab^ | 1.0±0.7^a^ | 7.5±2.1^a^ | 7.6±1.4^abcd^ |  | 0.3±0.2^a^ | 2.6±0.7^ab^ | 9.2±2.0^abc^ |
| terpinolene | 0^a^ | 0^ab^ | 1.4±0.9^ab^ | 5.1±1.5^a^ | 13.6±2.8^cd^ |  | 0.3±0.2^a^ | 5.3±1.8^ab^ | 14.1±2.3^c^ |
| camphor | 0.2±0.1^a^ | 0.9±0.6^a^ | 2.3±1.1^abc^ | 1.0±0.7^a^ | 14.4±4.9^cd^ |  | 0.1±0.1^a^ | 1.9±0.7^ab^ | 6.8±1.9^abc^ |
| sabinene | 0.1±0.1^a^ | 0.4±0.2^a^ | 0.7±0.2^a^ | 2.6±1.8^a^ | 10.3±2.4^cd^ |  | 0^a^ | 2.1±0.8^ab^ | 7.7±2.1^abc^ |
| *α*-terpineol | 0.2±0.1^a^ | 1.2±0.8^a^ | 8.3±3.8^bcd^ | 10.9±5.4^a^ | 17.2±3.3^cd^ |  | 0.5±0.3^a^ | 7.6±2.1^b^ | 15.0±2.8^c^ |
| *α*-terpinene | 0.1±0.1^a^ | 0.4±0.3^a^ | 3.4±2.4^abc^ | 6.8±2.6^a^ | 19.8±3.6^d^ |  | 0.3±0.2^a^ | 2.2±0.6^ab^ | 14.4±2.6^bc^ |
| *γ*-terpinene | 0.1±0.1^a^ | 0.5±0.3^a^ | 1.3±0.7^a^ | 9.6±4.8^a^ | 12.7±3.6^cd^ |  | 0.1±0.1^a^ | 4.7±1.6^ab^ | 13.2±2.7^bc^ |
| χ*^2^* | 34.717 | 178.33 | 238.54 | 69.951 | 3.8574 |  | 9.8377 | 90.91 | 77.726 |
| *p-*value | 0.0303 | <0.001 | <0.001 | <0.001 | <0.001 |  | 0.9809 | <0.001 | <0.001 |

^1^Number of OSNs tested.

^2^Spikes (Mean±SE) that were elicited by each test compound were compared using a generalized linear mixed model (GLMM) with a negative binomial distribution and log link function. Likelihood ratio tests (χ²) were used to assess the significance of fixed effects. Different letters in the same column indicate significant differences among compounds based on Šídák post hoc tests.

**Table S2.** Mean spike numbers (± SE) for SBaA and SBaB OSNs of *Monochamus* *saltuarius* responding to pheromones and host volatiles

| Compounds | SBaA | | | | |  | SBaB | | |
| --- | --- | --- | --- | --- | --- | --- | --- | --- | --- |
|  | A1 | A2 | A3 | A4 | A5 |  | B1 | B2 | B3 |
|  | 103^1^ | 13 | 3 | 6 | 6 |  | 39 | 38 | 27 |
| monochamol | 0 | 39.5±4.0^d2^ | 2.4±2.4^ab^ | 0^a^ | 7.0±7.0^a^ |  | 0.4±0.2 | 3.4±1.6^bcdefg^ | 9.7±2.1^abcdef^ |
| ipsenol | 0 | 3.9±0.8^bc^ | 35.2±3.8^c^ | 1.6±1.6^ab^ | 20.0±10.3^a^ |  | 0.3±0.2 | 7.9±2.6^gh^ | 15.6±4.5^cdef^ |
| ipsdienol | 0.1±0.1 | 3.2±1.1^bc^ | 31.6±6.0^c^ | 1.6±1.6^ab^ | 15.8±4.5^a^ |  | 0.3±0.2 | 6.2±2.1^efgh^ | 11.7±3.8^abcdef^ |
| (+)-*α*-pinene | 0 | 4.5±0.7^c^ | 0.4±0.4^ab^ | 0^a^ | 15.8±15.8^a^ |  | 0.4±0.2 | 8.8±2.7^b^ | 23.2±4.1^f^ |
| (-)-*α*-pinene | 0 | 4.5±0.8^c^ | 5.8±4.1^ab^ | 0^a^ | 21.8±17.3^a^ |  | 0.2±0.1 | 3.5±1.3^bcdefgh^ | 22.1±4.0^f^ |
| *β*-pinene | 0 | 4.7±0.7^c^ | 5.8±4.9^ab^ | 0^a^ | 12.3±0.9^a^ |  | 0.3±0.2 | 5.2±1.7^defgh^ | 18.5±3.2^def^ |
| (+)-limonene | 0 | 0.2±0.2^a^ | 0^a^ | 0^a^ | 5.0±2.9^a^ |  | 0.1±0.1 | 1.2±0.8^abc^ | 8.5±2.2^abcde^ |
| (-)-limonene | 0 | 0.2±0.2^a^ | 0^a^ | 0^a^ | 9.8±6.1^a^ |  | 0.3±0.2 | 1.5±0.8^abcde^ | 19.2±4.2^cdef^ |
| *β*-caryophyllene | 0 | 0.4±0.4^a^ | 0^a^ | 0^a^ | 4.5±2.6^a^ |  | 0.1±0.1 | 2.5±1.7^abcdef^ | 5.7±1.3^abc^ |
| myrcene | 0 | 0.5±0.5^a^ | 1.0±1.0^ab^ | 0^a^ | 12.5±7.5^a^ |  | 0 | 0.2±0.1^a^ | 5.3±1.4^ab^ |
| 3-carene | 0 | 0.3±0.3^a^ | 0^a^ | 0^a^ | 14.5±5.3^a^ |  | 0.1±0.1 | 2.3±0.9^abcdefg^ | 14.7±2.8^bcdef^ |
| ethanol | 0 | 0.2±0.2^a^ | 0^a^ | 0^a^ | 3.5±3.5^a^ |  | 0.2±0.2 | 0.2±0.1^a^ | 4.1±1.2^a^ |
| camphene | 0 | 0^a^ | 0^a^ | 0^a^ | 1.5±1.5^a^ |  | 0.3±0.2 | 0.4±0.2^ab^ | 3.6±0.9^a^ |
| *α*-phellandrene | 0 | 0^a^ | 0^a^ | 0^a^ | 15.0±9.4^a^ |  | 0.7±0.3 | 4.3±1.9^cdefgh^ | 18.8±4.8^cdef^ |
| ocimene | 0 | 0^a^ | 0^a^ | 0^a^ | 7.8±5.7^a^ |  | 0.5±0.3 | 4.5±2.2^cdefgh^ | 6.8±2.5^abcd^ |
| *p*-cymene | 0 | 0^a^ | 0^a^ | 0^a^ | 1.5±1.5^a^ |  | 0.5±0.3 | 1.5±0.7^abcde^ | 13.7±5.0^abcdef^ |
| terpinolene | 0 | 0^a^ | 0^a^ | 0^a^ | 8.5±6.1^a^ |  | 0.3±0.2 | 2.2±0.8^abcdefg^ | 18.2±3.4^ef^ |
| camphor | 0.1±0.1 | 1.2±0.8^ab^ | 8.4±3.5^bc^ | 7.4±3.0^bc^ | 16.3±3.6^a^ |  | 0.3±0.2 | 7.6±2.3^fgh^ | 14.7±5.0^bcdef^ |
| sabinene | 0 | 0.2±0.2^a^ | 0.6±0.4^ab^ | 0.4±0.4^a^ | 11.8±6.5^a^ |  | 0.1±0.1 | 1.0±0.3^abcd^ | 15.2±3.2^bcdef^ |
| *α*-terpineol | 0.1±0.1 | 1.8±1.0^abc^ | 10.4±7.1^bc^ | 24.4±6.3^c^ | 16.3±7.3^a^ |  | 0.5±0.3 | 4.8±1.4^cdefgh^ | 25.6±4.5^f^ |
| *α*-terpinene | 0 | 0.2±0.2^a^ | 3.6±3.6^ab^ | 1.0±0.8^ab^ | 12.5±9.0^a^ |  | 0 | 2.6±0.9^abcdefh^ | 15.7±3.9^bcdef^ |
| *γ*-terpinene | 0 | 0.5±0.2^a^ | 0^a^ | 2.4±2.2^ab^ | 6.3±4.0^a^ |  | 0.2±0.1 | 1.8±0.6^abcde^ | 11.9±3.8^abcdef^ |
| χ*^2^* | NA^3^ | 456.66 | 34.145 | 139.25 | 25.922 |  | NA | 167.21 | 114.71 |
| *p-*value | NA | <0.001 | <0.001 | <0.001 | 0.2094 |  | NA | <0.001 | <0.001 |

^1^Number of OSNs tested.

^2^Spikes (Mean±SE) that were elicited by each test compound were compared using a generalized linear mixed model (GLMM) with a negative binomial distribution and log link function. Likelihood ratio tests (χ²) were used to assess the significance of fixed effects. Different letters in the same column indicate significant differences among compounds based on Sidak post hoc tests.

^3^Not available


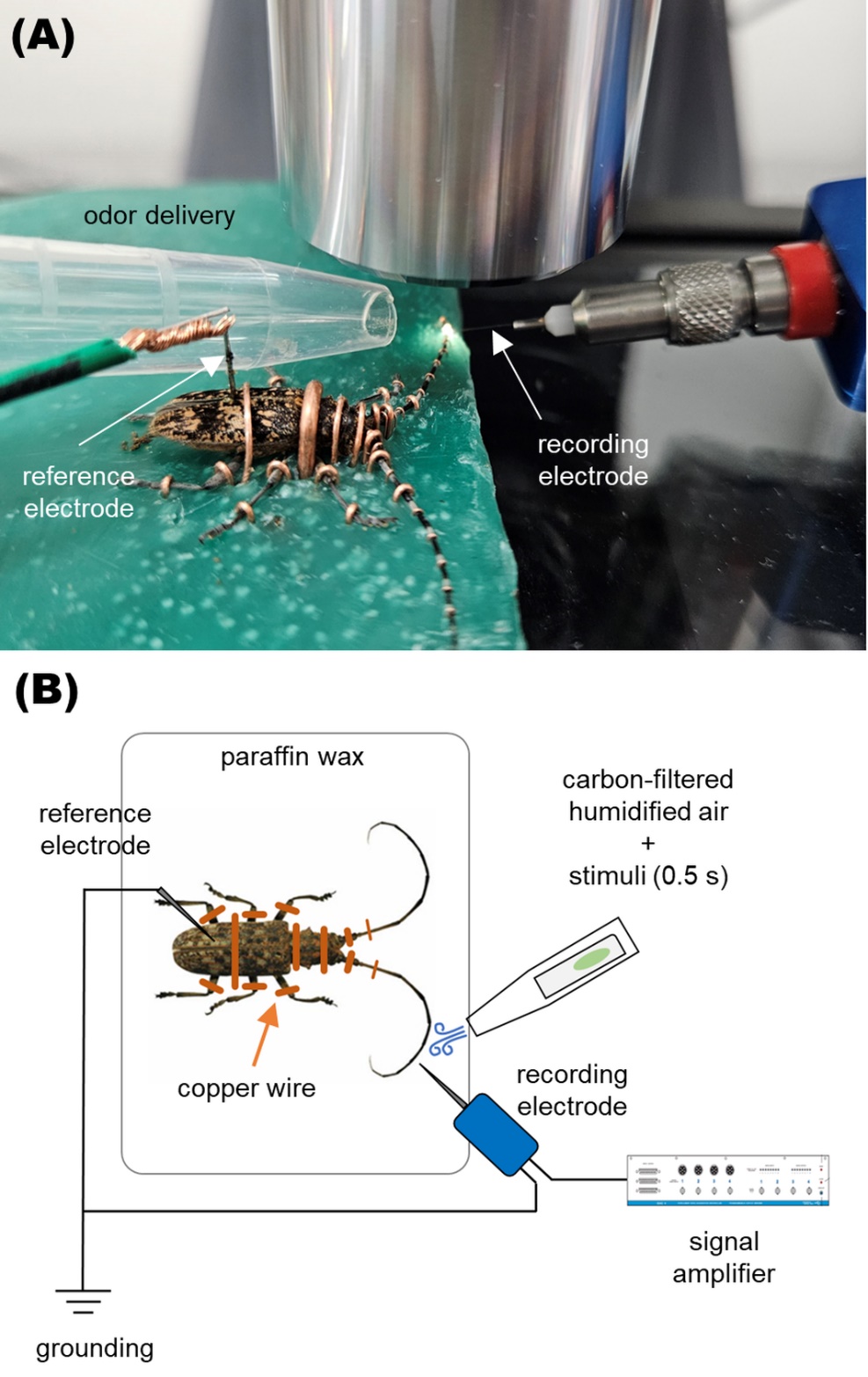


**Fig. S1.** Single sensillum recording (SSR) methods: schematic diagram of the setup (A); overview of preparation (B).


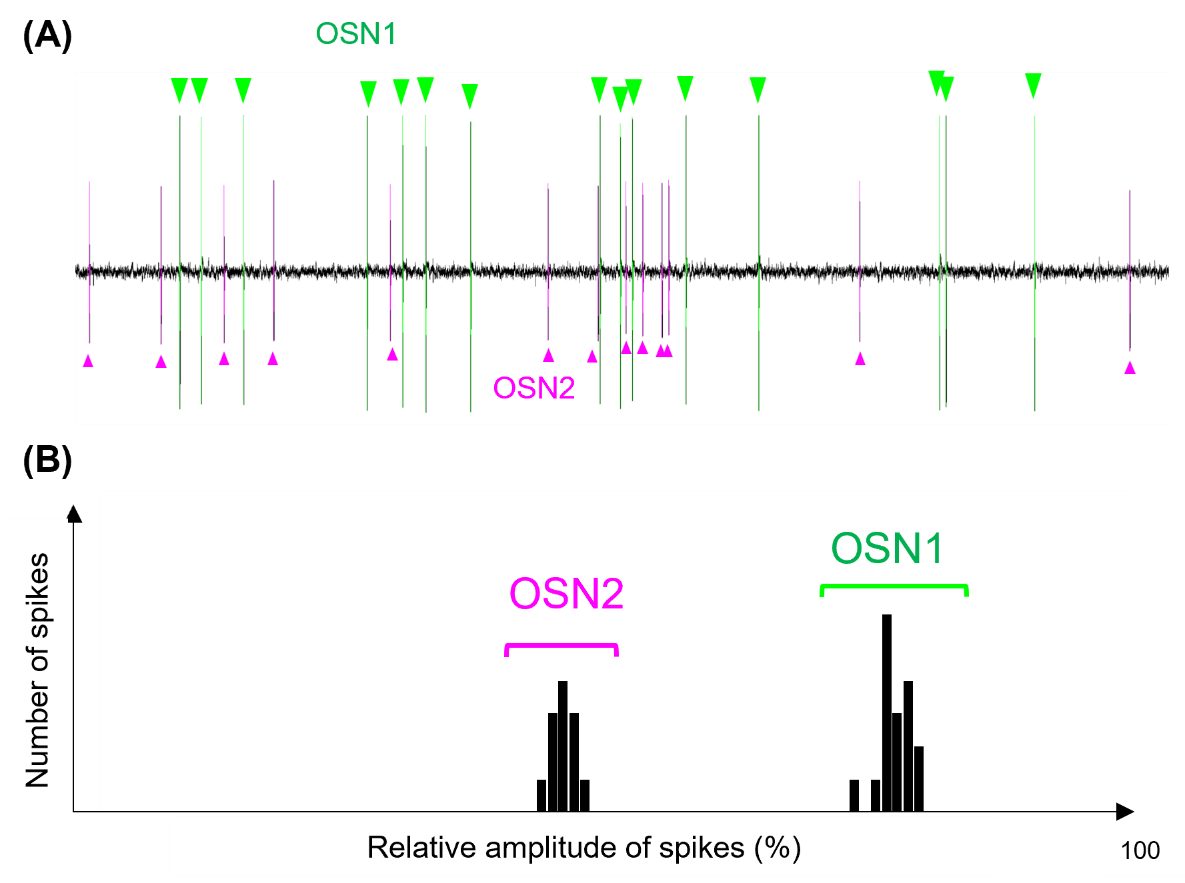


**Fig. S2.** Analysis of responses from a sensillum containing two olfactory sensory neurons (OSNs): The two OSNs (pink and green) are discriminated by differences in spike amplitude and waveform (A). Histogram of spike amplitudes with Gaussian distributions illustrating separation of the two OSNs (B).


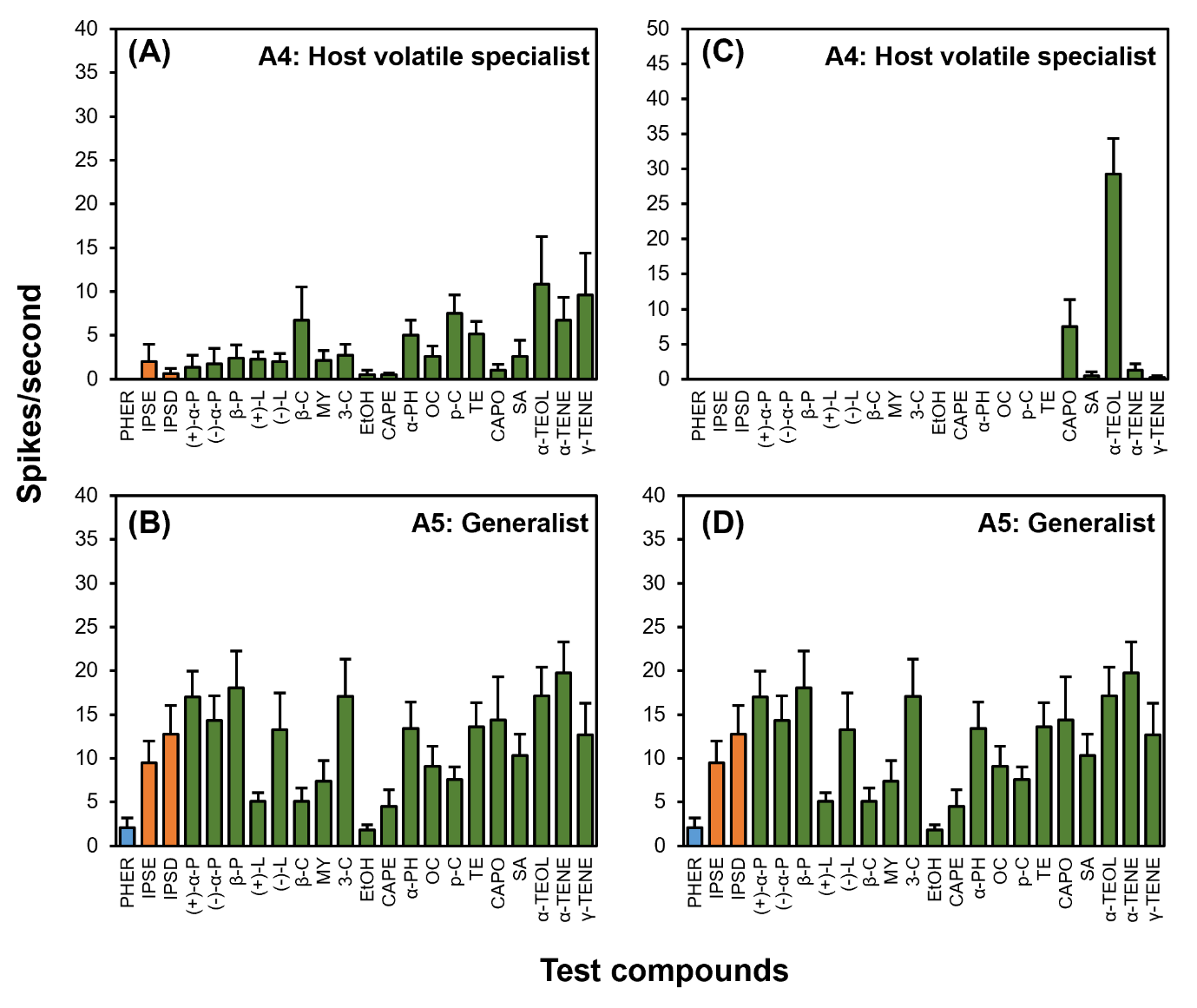


**Fig. S3.** Mean response of A4 and A5 OSN types to 100 μg of each test compound in *Monochamus alterantus* (A and B) and in *M*. *saltuarius* (C and D). Colors denote volatile categories (blue, aggregation-sex pheromone; orange, bark beetle pheromone; green, host volatiles; abbreviations as in Table 1). Mean spike numbers (±SE) and statistics are provided in **Supplementary Tables S1**–**S2**.
